# Rapidly Progressed Distal Arch Aneurysm with Distal Open Stent Graft-Induced New Entry Caused by “Spring-Back” Force

**DOI:** 10.3400/avd.cr.20-00075

**Published:** 2020-09-25

**Authors:** Masami Takagaki, Hirofumi Midorikawa, Hiroki Yamaguchi, Hiromasa Nakamura, Tasuku Kadowaki, Yousuke Ueno, Takaki Uchida, Tomoyuki Aoki

**Affiliations:** 1 Department of Cardiovascular Surgery, Showa University Koto Toyosu Hospital; 2 Department of Cardiovascular Surgery, Southern Tohoku General Hospital

**Keywords:** stent graft-induced new entry, spring-back force, open stent graft

## Abstract

The J Graft Open Stent Graft (JOSG) is used for the frozen elephant trunk procedure in Japan. We report a 70-year-old male who developed a rapidly progressing distal arch aneurysm caused by a distal stent graft-induced new entry (DSINE) 7 months after the procedure. The JOSG was originally implanted at the curved part of the distal arch. It created its initial DSINE on the greater curve and rapidly “sprang” back in 2 months. Urgent thoracic endovascular aortic repair fixed this serious complication. We should remember such rapid progression of DSINE by JOSG and treat its initial sign earlier.

## Introduction

Total arch replacement using the frozen elephant trunk procedure has been accepted as a standard technique for thoracic aortic arch aneurysm or dissection.^1,2)^ However, a distal stent graft-induced new entry (DSINE), defined as a new tear caused by the stent graft at its distal end, can be life-threatening especially in type B aortic dissection.^3)^ The stent graft, like a spring, has an inherent tendency to “spring-back” to its initial straight position when passively bent at the aortic arch, thus generating stress on the greater curve, especially at its ends. This stress is called the “spring-back” force.^4,5)^ The J Graft Open Stent Graft (JOSG, Japan Lifeline Co., Ltd., Tokyo, Japan), which is currently the only commercially available device in Japan used in conducting the frozen elephant trunk technique,^6,7)^ has a considerable spring-back force. We hereby report a typical case of DSINE caused by the spring-back force of this device after the treatment of true saccular arch aneurysm. DSINE rapidly progressed to distal arch aneurysm in 2 months. Although the aneurysm was successfully treated by thoracic endovascular aortic repair (TEVAR), we should remember that such rapid progression of DSINE to the aneurysm may occur at any time.

## Case Report

**Figure 1:**
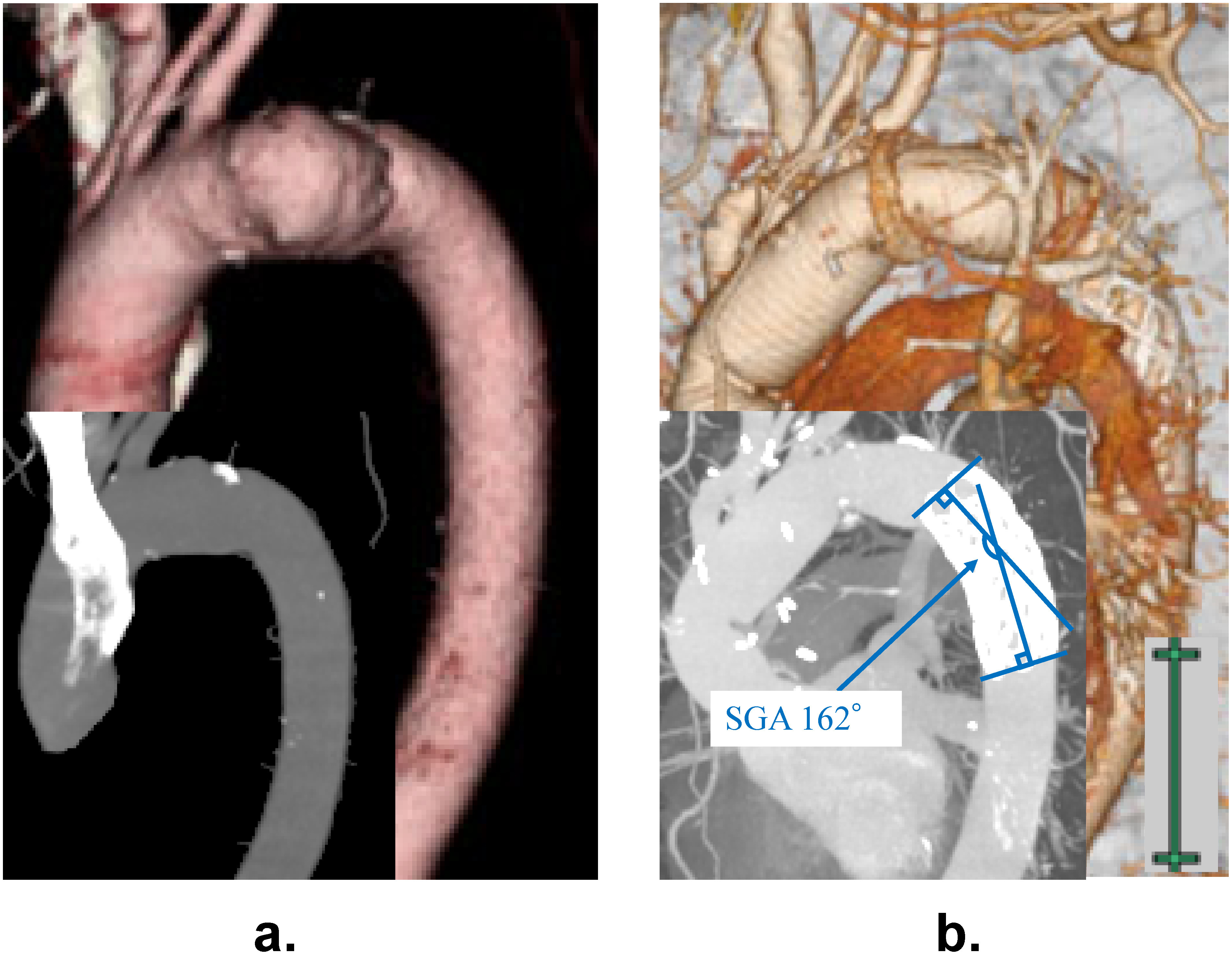
Fig. 1 Pre- and post-operative CT images with three-dimensional reconstruction before and after the total arch replacement using the frozen elephant trunk technique. (**a**) Preoperative CT image showing a saccular-type aortic arch aneurysm. The size of the descending thoracic aorta was 26.5 mm at Th6 and gradually tapered to 24.4 mm at Th8. (**b**) Postoperative CT image showing the total arch replacement using the frozen elephant trunk procedure with 27-mm JOSG. The SGA was 162° at this time.

CT: computed tomographic; JOSG: J Graft Open Stent Graft; SGA: stent graft angle defined as an angle between lines perpendicular to the proximal and distal stumps in the left anterior oblique 60° view of three-dimensional CT angiography

A 70-year-old man had a recent total arch replacement with the frozen elephant trunk technique using a 27-mm JOSG for his true saccular arch aneurysm (**Fig. 1a**). He was considered a candidate for definitive open repair because he was young and did not have significant past medical issues. The descending aorta at the distal landing zone for the JOSG was 26.5 mm at Th6 and gradually tapered in diameter to 24.4 mm at Th8, and the JOSG was not oversized. To evaluate how much the stent graft was bent at implantation, we measured the stent graft angle (SGA), which is defined as an angle between lines perpendicular to proximal and distal stent graft stumps in the left anterior oblique 60° view of the three-dimensional computed tomographic (CT) angiography. The JOSG was originally placed in the curved part of the distal arch and mildly bent with its SGA of 162° (**Fig. 1b**). The patient’s postoperative course was uneventful, and he was being followed up at our clinic.

**Figure 2:**
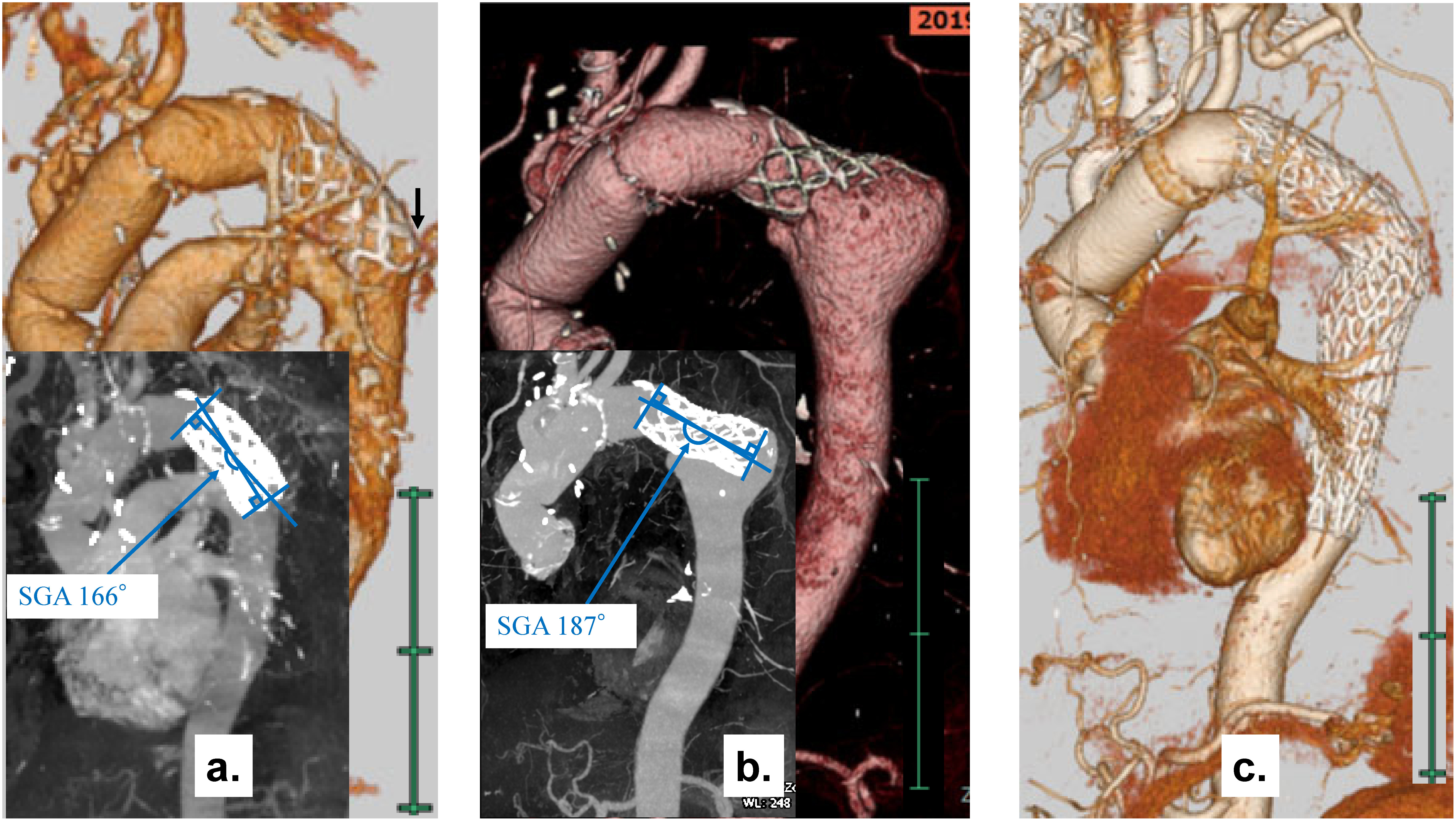
Fig. 2 Follow-up CT images with three-dimensional reconstruction after the total arch replacement with the frozen elephant trunk procedure. (**a**) Five months after the surgery. A small DSINE (arrow) appeared in the distal end of the JOSG at the greater curvature of the descending aorta. The SGA was slightly increased to 166°. (**b**) Seven months after the surgery. Rapid progression of DSINE and aneurysm in the descending aorta were shown, and the stent graft was straightened to the SGA of 187°. (**c**) Post-procedural CT showed that the DSINE disappeared after thoracic endovascular aortic repair.

CT: computed tomographic; DSINE: distal stent graft-induced new entry; JOSG: J Graft Open Stent Graft; SGA: stent graft angle, defined as an angle between lines perpendicular to proximal and distal stumps in the left anterior oblique 60° view of three-dimensional CT angiography

He suddenly developed hemoptysis 5 months after his surgery. CT angiography showed a small DSINE at the greater curvature of the JOSG, which seemed typical of the “spring-back” force origin. The SGA had slightly increased to 166° (**Fig. 2a**). However, his bronchography revealed that his bleeding came from the right upper lobe. Therefore, DSINE was unlikely to be the cause of the hemoptysis. Furthermore, the patient’s exercise capacity was moderately affected (modified Rankin scale 3). We prioritized his rehabilitation. He was managed medically and transferred to a rehabilitation hospital with no further episodes. However, after 2 months, he developed hemoptysis again. CT angiography showed a rapid progression of DSINE with a large aneurysm at the descending aorta (**Fig. 2b**). The stent graft was straightened with SGA of 187° showing the spring-back force of the device. Urgent TEVAR was successfully performed using comfortable TAG (W. L. Gore & Associates, Flagstaff, AZ, USA). Post-procedural CT angiography showed a diminished distal arch aneurysm without any endoleak (**Fig. 2c**). The patient recovered uneventfully and has been doing well without further episodes of hemoptysis. He is currently being followed up at our clinic.

## Discussion

The purpose of this report is to demonstrate that a typical DSINE caused by the spring-back force of the JOSG rapidly progressed in 2 months and to recommend earlier endovascular intervention at its initial sign. The JOSG consists of a woven structure of Nitinol wire and a superelastic shape-memory alloy, which can achieve adequate radial force to fix the device to the vessel.^6)^ However, at the same time, this device can produce considerable spring-back force if deployed in the curved portion. We judged our case to be DSINE caused by spring-back force for two reasons. First, we identified an initial injury at the greater curvature of descending aorta, precisely at the distal end of the device, where the stress by the force was generated. Second, we induced a parameter of SGA, which increased from 162° to 166° at the initial injury and completely straightened at 187° after aneurysm progression. We think that the SGA increase observed at the follow-up indicates the spring-back force to straighten the stent graft. In our experience, we found five DSINE cases out of 48 during a mean follow-up of 575 days. Pre- and post-procedural three-dimensional CT angiographies were available in 17 cases, and the SGA significantly increased from 152.5°±19.2° to 157.9°±20.8° (P=0.005). Consequently, we now consider an initial small DSINE with a SGA increase to be an indication for early intervention to prevent a further catastrophic aortic event.

Our case was initially a true aneurysm of the aortic arch. Yamane et al. noted the possibility of aorto-esophageal fistula caused by DSINE after implantation of this device in aortic dissection.^8)^ To date, DSINE by other devices has also been reported after the frozen elephant trunk procedure.^3,9)^ Our case report suggests that this type of DSINE can occur even after the repair of a true aneurysm.

Li et al. investigated the risk factors for DSINE in their type B aortic dissection patients and found that a stent graft length of less than 145 mm was a significant risk factor for DSINE.^5)^ They speculated that the shorter the stent graft, the stronger the spring-back force at the greater curvature of the distal end. We had implanted a 60-mm-long JOSG. Because the JOSG has a length-adjustable non-stent part, trimming the non-stent part long enough to land the stent part at the straight descending thoracic aorta is a key to avoiding DSINE. However, care must be taken to avoid causing paraplegia, which is known to increase with extensive coverage of the thoracic aorta. Preoperative identification of the Adamkiewicz artery would be helpful in determining the safe length of the non-stent part of the JOSG before implantation. Although the stent graft was short (60 mm) in our case, its position did not look bad with its distal end placed in the straight descending aorta (**Fig. 1b**). Because the JOSG was not oversized in this case, a lack of radial force may be related to the movement of the JOSG during rapid progression.

## Conclusion

We present a case of a small DSINE caused by spring-back force after the repair of a true aneurysm using JOSG implantation, which rapidly progressed to the distal arch aneurysm. Early additional endovascular intervention is recommended even in such small DSINE.
